# Supplementary material for: VT68.2: An Antibody to Chondroitin Sulfate Proteoglycan 4 (CSPG4) Displays Reactivity against a Tumor-Associated Carbohydrate Antigen
Source: Int J Mol Sci. 2023 Jan 28;24(3):2506. doi: 10.3390/ijms24032506 (PMC9917008; doi:10.3390/ijms24032506)

## Slide 1
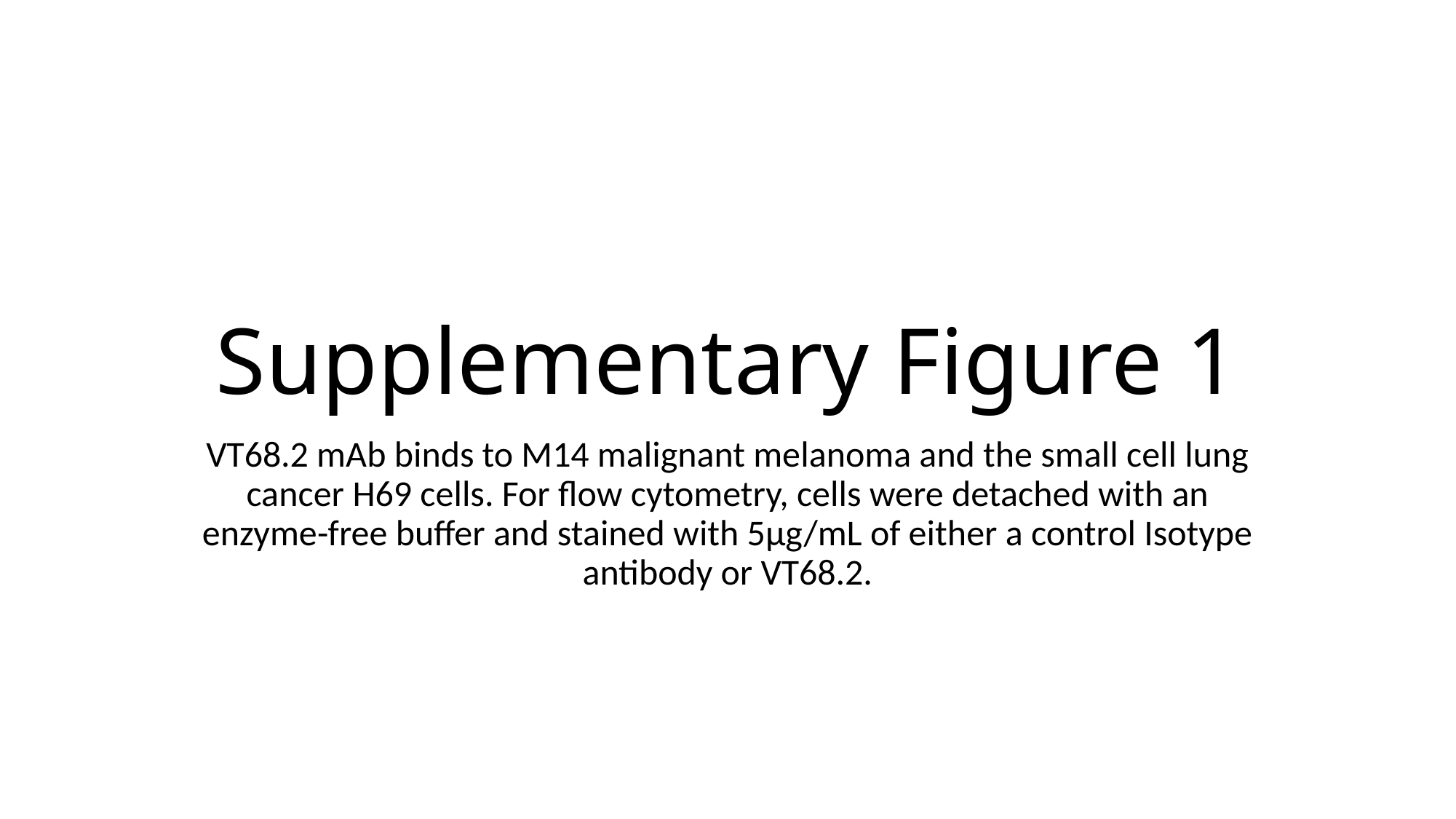

# Supplementary Figure 1
VT68.2 mAb binds to M14 malignant melanoma and the small cell lung cancer H69 cells. For flow cytometry, cells were detached with an enzyme-free buffer and stained with 5µg/mL of either a control Isotype antibody or VT68.2.

## Slide 2
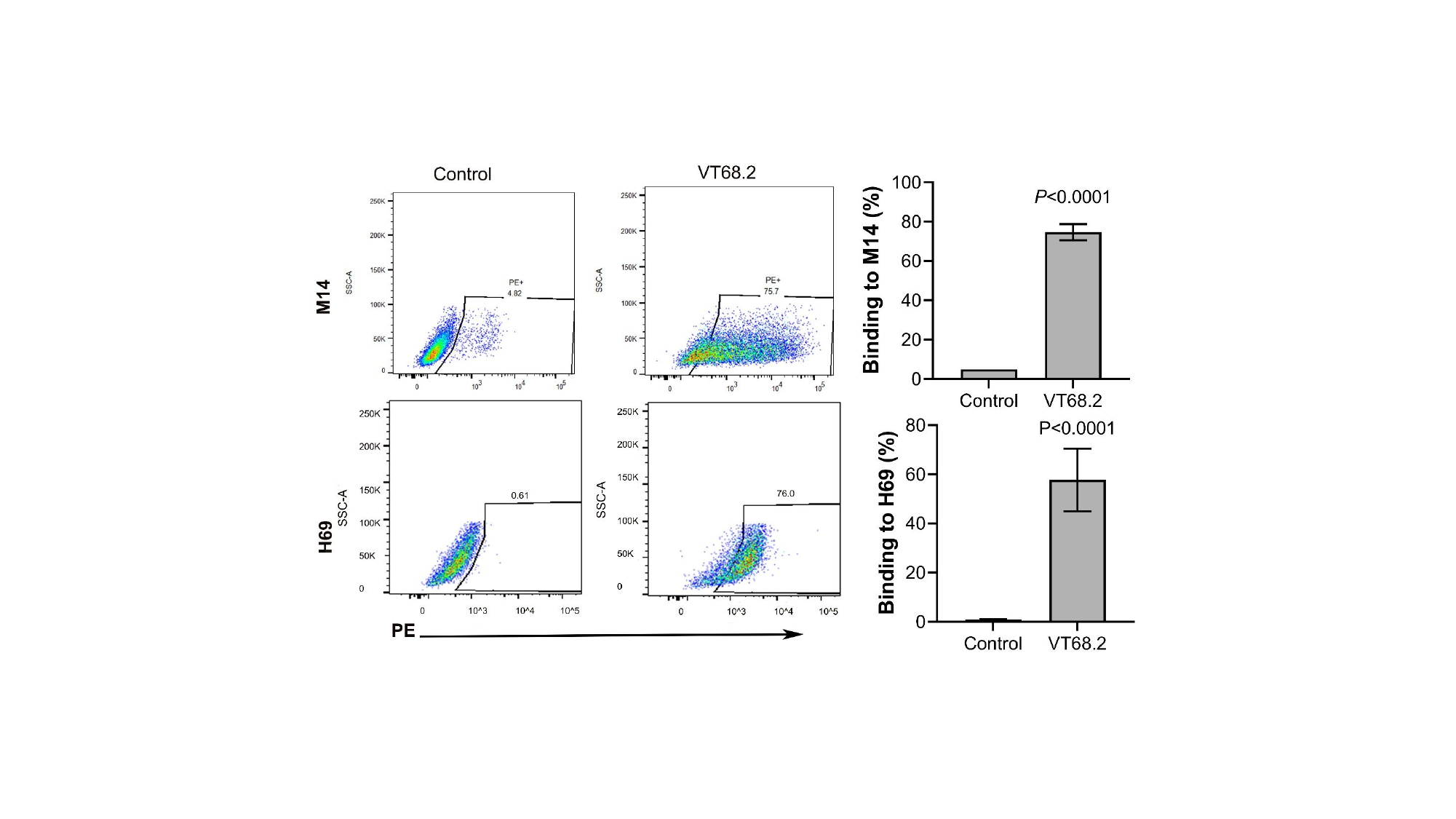

Supplement: Supplementary file 1 [file ijms-24-02506-s001.zip › Supplementary Figure S1.pptx]
