# Supplementary material for: VT68.2: An Antibody to Chondroitin Sulfate Proteoglycan 4 (CSPG4) Displays Reactivity against a Tumor-Associated Carbohydrate Antigen
Source: Int J Mol Sci. 2023 Jan 28;24(3):2506. doi: 10.3390/ijms24032506 (PMC9917008; doi:10.3390/ijms24032506)
Supplement: Supplementary file 1 [file ijms-24-02506-s001.zip › Supplementary Figure S2.pptx]

## Slide 1
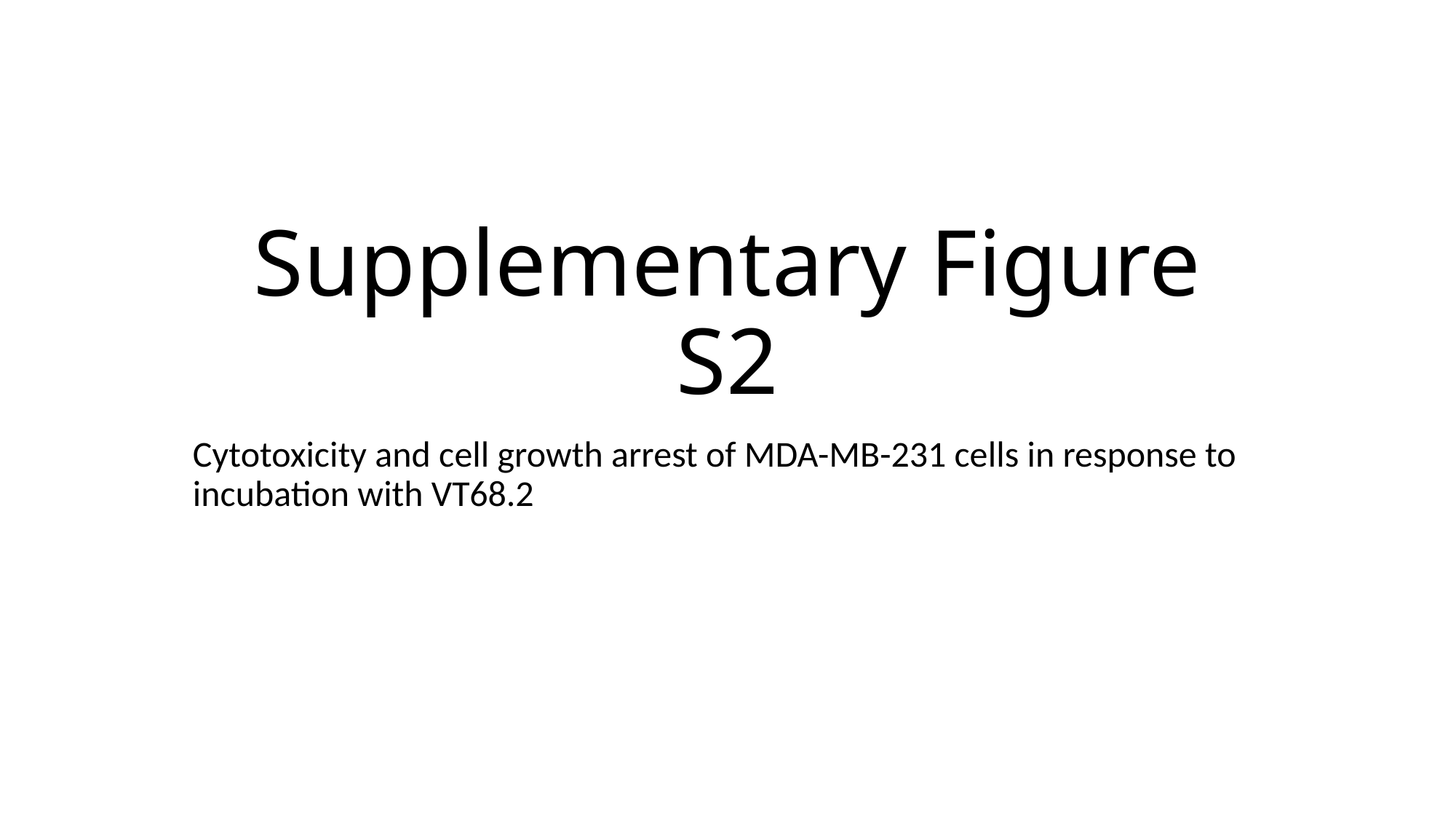

# Supplementary Figure S2
Cytotoxicity and cell growth arrest of MDA-MB-231 cells in response to incubation with VT68.2

## Slide 2
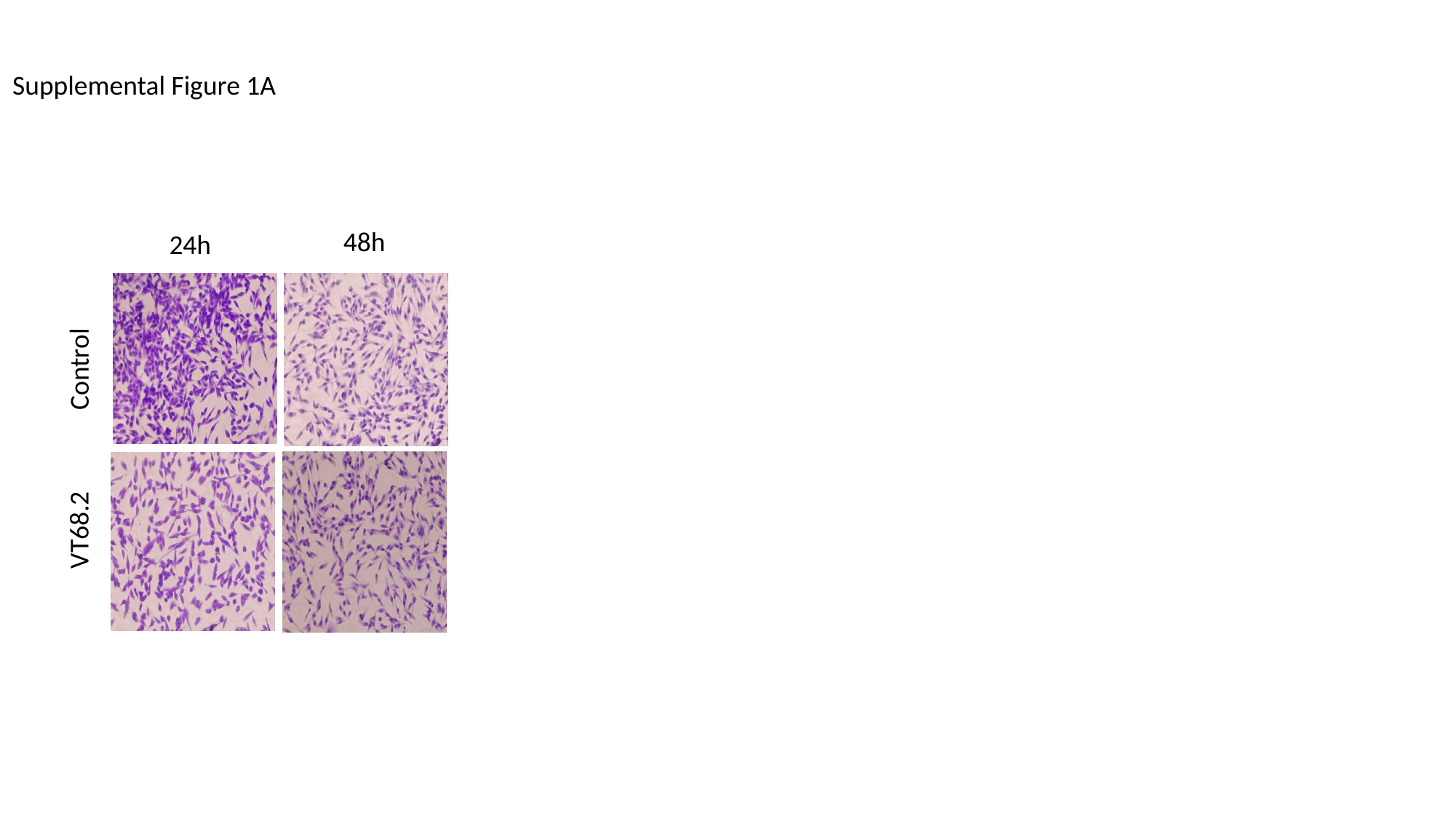

Supplemental Figure 1A
48h
24h
Control
VT68.2

## Slide 3
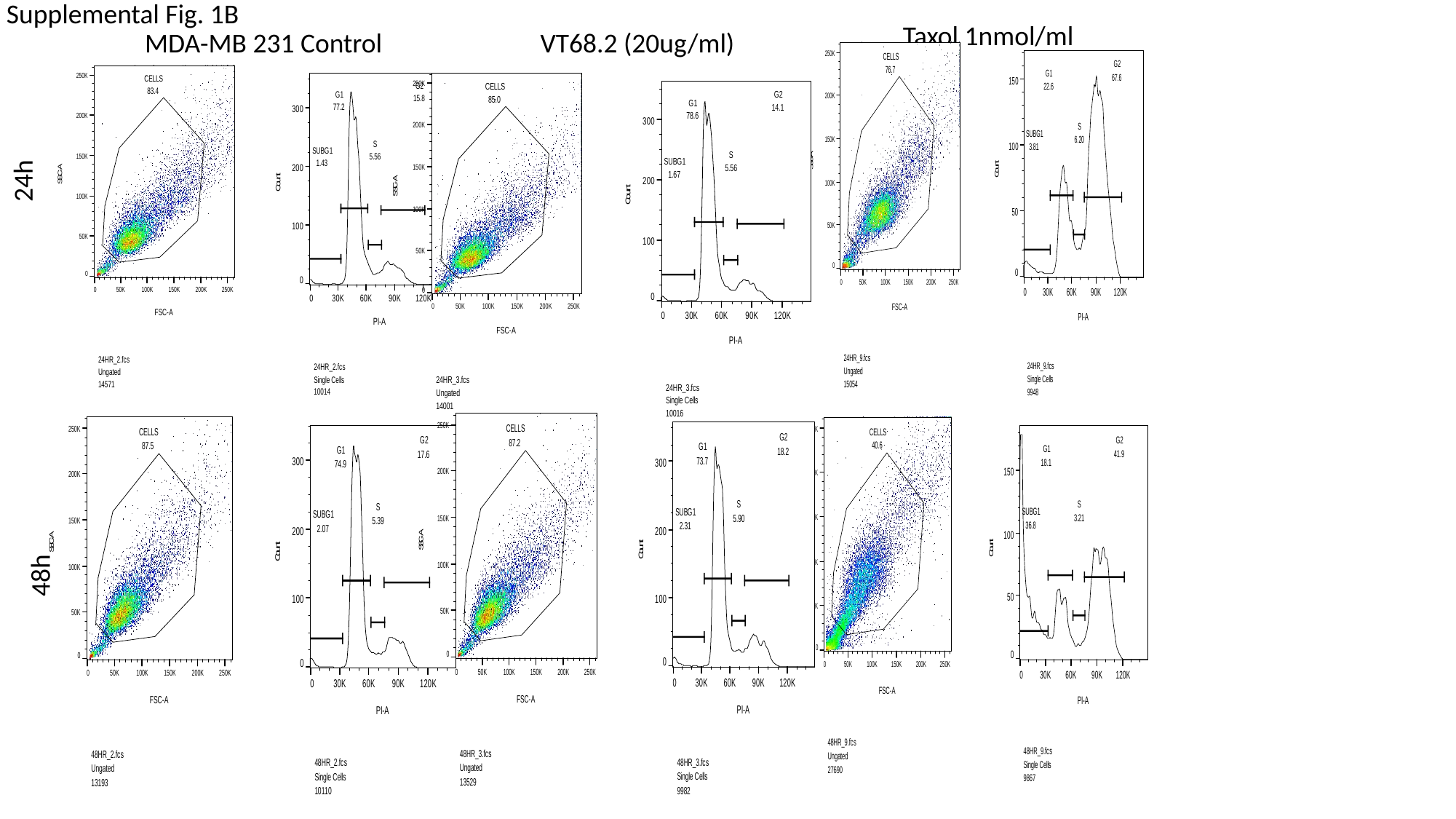

Supplemental Fig. 1B
Taxol 1nmol/ml
MDA-MB 231 Control
VT68.2 (20ug/ml)
24h
48h
